# Blood Cell Biomarkers of Inflammation and Cytokine Levels as Predictors of Response to Biologics in Patients with Psoriasis

**DOI:** 10.3390/ijms24076111

**Published:** 2023-03-24

**Authors:** Clara Sophie Bramsen Andersen, Amanda Kvist-Hansen, Mie Siewertsen, Christian Enevold, Peter Riis Hansen, Diljit Kaur-Knudsen, Claus Zachariae, Claus Henrik Nielsen, Nikolai Loft, Lone Skov

**Affiliations:** 1 Department of Dermatology and Allergy, Herlev and Gentofte Hospital, Copenhagen University Hospital, 2900 Hellerup, Denmark; 2 Institute for Inflammation Research, Center for Rheumatology and Spine Diseases, Rigshospitalet, Copenhagen University Hospital, 2200 Copenhagen, Denmark; 3 Department of Cardiology, Herlev and Gentofte Hospital, Copenhagen University Hospital, 2900 Hellerup, Denmark; 4 Department of Clinical Medicine, Faculty of Health and Medical Sciences, University of Copenhagen, 2200 Copenhagen, Denmark; 5 Department of Odontology, Faculty of Health and Medical Sciences, University of Copenhagen, 2200 Copenhagen, Denmark

**Keywords:** psoriasis, biologics, response to treatment, predictive biomarkers, blood cytokines, neutrophil-to-lymphocyte ratio

## Abstract

For people with psoriasis, biomarkers aiding in the personalization of treatment with biologics are needed. We examined the usefulness of several biomarkers of inflammation in this respect. The neutrophil-to-lymphocyte ratio (NLR), the platelet-to-lymphocyte ratio (PLR), and the systemic immune–inflammation index (SII) were measured in patients with psoriasis initiating TNF-α inhibitors (*n* = 131), IL-17/IL-17R inhibitors (*n* = 65), or IL-23/IL-12/23 inhibitors (*n* = 50). The blood levels of tumor necrosis factor (TNF)-α, interleukin (IL)-1β, interferon (IFN)-γ, IL-17A, IL-6, soluble IL-6 receptor (sIL-6R), and soluble IL-6 signal transducer (sIL-6ST) were measured in patients initiating adalimumab (*n* = 62) or IL-17/IL-17R inhibitors (*n* = 24). Treatment response was defined by a psoriasis area and severity index (PASI) ≤ 2 three months after treatment initiation. Responders to TNF-α inhibitors had a lower NLR at baseline than non-responders (median and interquartile range (IQR) 2.15 (1.67–2.86) vs. 2.54 (1.88–3.55); *p* = 0.04). Responders to treatment with adalimumab had lower IL-6 levels at baseline than non-responders (0.99 (0.42–1.4) vs. 1.62 (0.96–2.41) pg/mL; *p* = 0.02). For the majority of patients, the IL-17A, IL-1β, and IFN-γ levels were below quantification limits. NLR and IL-6 may serve as predictive biomarkers of treatment response to TNF-α inhibitor therapy in patients with psoriasis.

## 1. Introduction

Psoriasis is a chronic inflammatory disease with a major influence on quality of life [1]. The pathogenesis of psoriasis is characterized by the activation of both innate and adaptive immune responses in the skin. Innate immune cells release pro-inflammatory cytokines, such as the tumor necrosis factor (TNF)-α, interferon (IFN)-γ, and interleukin (IL)-1β, and these cytokines facilitate the activation of myeloid dendritic cells, which release IL-12 and IL-23, which are crucial for the differentiation of naive T-cells into T_h_1, T_h_22, and T_h_17 subsets [2]. TNF-α, IFN-γ, IL-22, and IL-17 produced by these Th cell subsets sustain the inflammatory process and induce the proliferation of keratinocytes. Biologics targeting these cytokines or their receptors are used for the treatment of moderate to severe psoriasis. However, not all patients respond to this treatment, and some biologics lose their treatment effect over time.

Today, guidelines based on disease severity and drug cost-effectiveness advise on when a patient is eligible for biologic treatment and which biologics should be used [3]. However, there is limited knowledge about which biologics will be effective for an individual patient. This practice of trial-and-error is both costly and demotivating for the patients, and there is a need for predictive biomarkers before personalized treatment can become a reality. Several candidate biomarkers have been described, but at present, none are suitable for clinical use without further validation [4]. For instance, an association between carrying the HLA-C*06:02 allele and a favorable response to ustekinumab has been described [5]. Moreover, some clinical features are known to affect treatment response, such as being overweight and smoking, which both decrease the chances of a favorable treatment response [6,7,8]. Ideally, biomarkers should have high sensitivity and specificity, and they should be easy to implement clinically. Biomarkers of systemic inflammation obtained from standard blood tests, such as proinflammatory cytokines, C-reactive protein (CRP), the neutrophil-to-lymphocyte ratio (NLR), the platelet-to-lymphocyte ratio (PLR), and the systemic immune–inflammation index (SII), have gained interest in this respect. All of these biomarkers have been shown to be increased in patients with psoriasis when compared to healthy controls [9,10,11,12], and some have been associated with disease severity and cardiovascular disease in psoriasis [13,14,15].

In this study, we investigated the blood cell biomarkers of inflammation and cytokines at baseline (before initiation of biologic treatment) to determine if any of these biomarkers hold potential to predict treatment response to biologics.

## 2. Results

### 2.1. Characteristics of Study Population—Blood Cell Biomarkers of Inflammation

The analyses were performed on data from 236 patients, and included the following 246 treatment series: 131 with TNF-α inhibitors (adalimumab *n* = 125, infliximab *n* = 3, certolizumab pegol *n* = 2, and etanercept *n* = 1), 65 with IL-17 inhibitors (secukinumab *n* = 31 and ixekizumab *n* = 21), or IL-17R inhibitor (brodalumab *n* = 13), and 50 with IL-23/IL-12/23 inhibitors (guselkumab *n* = 10, risankizumab *n* = 3, and ustekinumab *n* = 37) (Figure S1 and Table S1). Most patients shifted directly from a conventional systemic anti-psoriatic drug to a biologic drug or from one biologic drug to another. The TNF-α inhibitor group included 73 (60 bio-naive) responders and 58 (48 bio-naive) non-responders, the IL-23/IL-12/23 inhibitor group included 29 (25 bio-naive) responders and 21 (10 bio-naive) non-responders, and the IL-17/IL17R inhibitor group included 54 (23 bio-naive) responders and 11 (1 bio-naive) non-responders, respectively.

In the IL-17/IL-17R inhibitor group, the proportion of bio-naive non-responders was low (*n* = 1) and did not allow for further analysis. The baseline characteristics and levels of NLR, PLR, and SII for responders and non-responders according to the class of biologics are reported in Table 1.

### 2.2. Responders to TNF-α Inhibitors Had Lower NLR at Baseline Than Non-Responders

**Figure 1:**
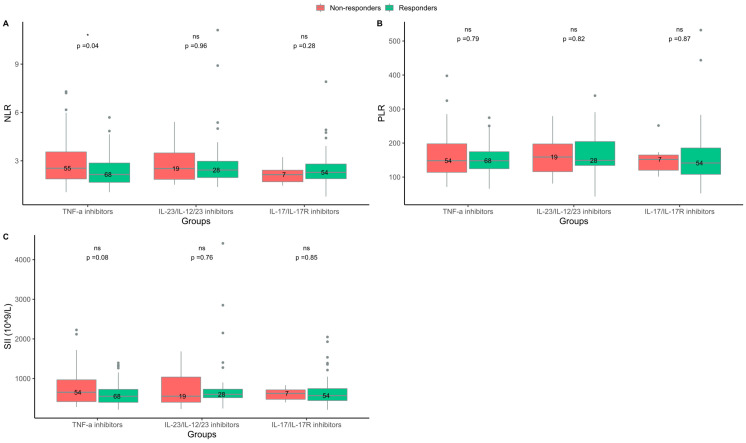
Blood cell biomarkers at baseline in responders and non-responders treated with either tumor necrosis factor (TNF)-α inhibitors, interleukin (IL)-23/IL-12/23 inhibitors, or IL-17/IL-17R inhibitors. (**A**) Neutrophil-to-lymphocyte ratio (NLR). (**B**) Platelet-to-lymphocyte ratio (PLR). (**C**) Systemic immune–inflammation index (SII). Responders = psoriasis area and severity index (PASI) ≤ 2 three months after treatment start. Non-responders = PASI > 2 three months after treatment start. ns = non-significant. * *p* < 0.05.

**Figure 2:**
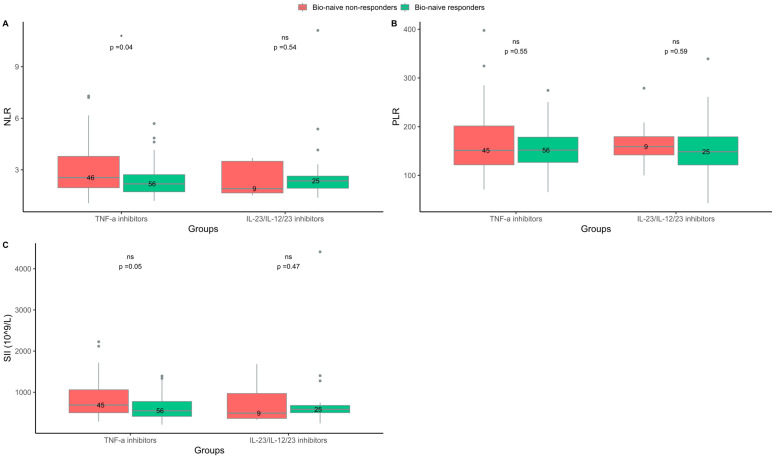
Blood cell biomarkers at baseline in bio-naive responders and non-responders treated with either tumor necrosis factor (TNF)-α inhibitors or interleukin (IL)-23/IL-12/23 inhibitors. (**A**) Neutrophil-to-lymphocyte ratio (NLR). (**B**) Platelet-to-lymphocyte ratio (PLR). (**C**) Systemic immune–inflammation index (SII). Responders = psoriasis area and severity index (PASI) ≤ 2 three months after treatment start. Non-responders = PASI > 2 three months after treatment start. ns = non-significant. * *p* < 0.05.

In the group treated with TNF-α inhibitors, the median (IQR) NLR at baseline was lower in responders than in non-responders (2.2 (1.7–2.9) vs. 2.5 (1.9–3.6); *p* = 0.04), (Figure 1). NLR was also lower in bio-naive responders compared to bio-naive non-responders (2.2 (1.7–2.7) vs. 2.6 (2.0–3.8); *p* = 0.04), (Figure 2). Further testing by logistic regression confirmed that lower values of NLR at baseline were associated with a response to TNF-α inhibitor treatment in both an unadjusted analysis and an analysis including adjustments for BMI, smoking status, and baseline PASI (Table 2. No difference was found between responders and non-responders to TNF-α inhibitor treatment regarding SII or PLR at baseline (Figure 1 and Figure 2).

In the groups treated with IL-23/IL-12/23 inhibitors or IL-17/IL-17R inhibitors, there were no differences in NLR, SII, and PLR at baseline between responders and non-responders (Figure 1 and Figure 2).

In sub-analyses where the treatment response was defined by achievement of PASI ≤ 4, there was no difference between the baseline levels of NLR, PLR, or SII in responders compared to non-responders in any of the treatment groups (Tables S2 and S5, Figures S3 and S4). However, with this less restrictive definition of treatment response, there were few non-responders in each treatment group (Table S3).

### 2.3. Characteristics of the Study Population—Blood Cytokines

A total 80 patients and 86 treatment series were included in the analysis, 62 with the TNF-α inhibitor adalimumab and 24 with IL-17/IL-17R inhibitors (ixekizumab *n* = 11, secukinumab *n* = 1, and brodalumab *n* = 12), (Figure S2). The adalimumab group included 35 (31 bio-naive) responders and 27 (24 bio-naive) non-responders. The IL-17/IL-17R inhibitor group included 17 responders and 7 non-responders, and all the patients were bio-experienced. The baseline characteristics and cytokine levels of the responders and non-responders according to the class of biologics are reported in Table 3.

### 2.4. Levels of IL-6 and sIL-6R Were Higher in Non-Responders to the TNF-α Inhibitor Adalimumab

**Figure 3:**
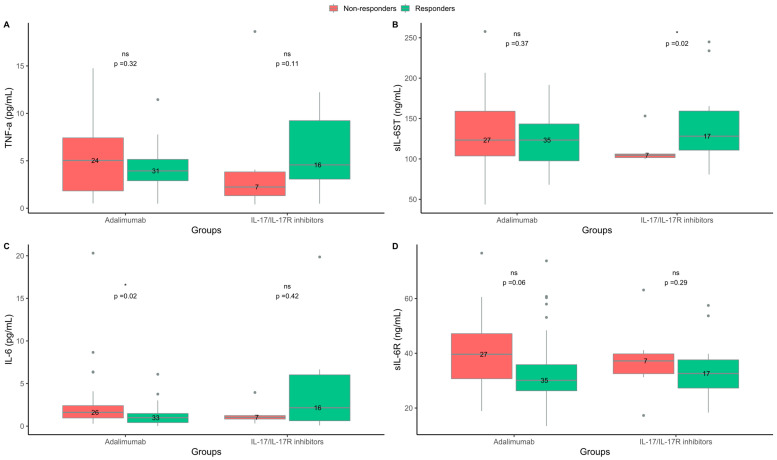
Blood cytokine levels at baseline in responders and non-responders to treatment with adalimumab or interleukin (IL)-17/IL-17R inhibitors. (**A**) Tumor necrosis factor (TNF)-α. (**B**) Soluble interleukin-6 signal transducer (sIL-6ST). (**C**) Interleukin-6 (IL-6). (**D**) Soluble interleukin-6 receptor (sIL-6R). Responders = psoriasis area and severity index (PASI) ≤ 2 three months after treatment start. Non-responders = PASI > 2 three months after treatment start. ns = non-significant. * *p* < 0.05.

**Figure 4:**
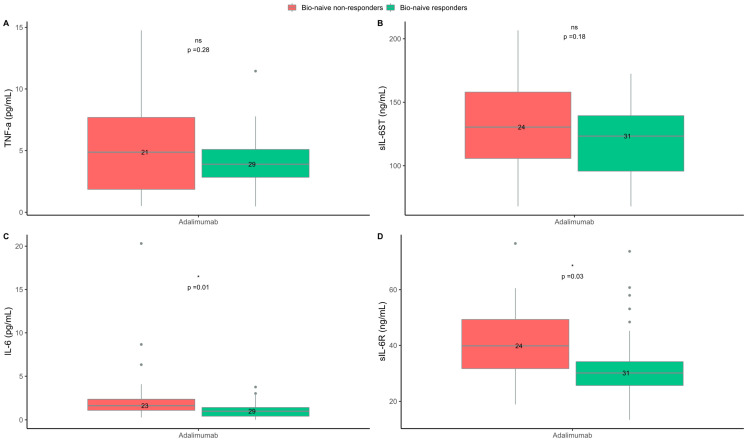
Blood cytokine levels at baseline in bio-naive responders and non-responders to treatment with adalimumab. (**A**) Tumor necrosis factor (TNF)-α. (**B**) Soluble interleukin-6 signal transducer (sIL-6ST). (**C**) Interleukin-6 (IL-6). (**D**) Soluble interleukin-6 receptor (sIL-6R). Responders = psoriasis area and severity index (PASI) ≤ 2 three months after treatment start. Non-responders = PASI > 2 three months after treatment start. ns = non-significant. * *p* < 0.05.

In the adalimumab group, the median (IQR) level of IL-6 was lower at baseline in responders than in non-responders (1.0 (0.4–1.5) vs. 1.6 (1.0–2.4) pg/mL; *p* = 0.02). Moreover, the levels of sIL-6R were lower in responders than non-responders (30.1 (26.3–35.8) vs. 39.6 (30.7–47.2) pg/mL; *p* = 0.06), (Figure 3). The levels of IL-6 and sIL-6R were also lower when only investigating bio-naive responders compared to bio-naive non-responders (1.0 (0.4–1.4) vs. 1.6 (1.1–2.4) pg/mL; *p* = 0.01 and 30.1 (25.6–34.2) vs. 39.8 (31.7–49.3) ng/mL; *p* = 0.03, respectively), (Figure 4). In the logistic regression analysis, lower baseline levels of IL-6 were also associated with the response to adalimumab; however, the association between the levels of sIL-6R and the response to adalimumab in the bio-naive subgroup was not confirmed in the logistic regression analysis (Table 2). In the adalimumab group, there was no difference in the baseline levels of TNF-α and sIL-6ST between responders and non-responders. In the IL-17/IL-17R inhibitor group, sIL-6ST was higher at baseline in responders than in non-responders (128 (111–159) vs. 105 (102–106) ng/mL; *p* = 0.02), (Figure 3). However, in the logistic regression analysis, no association between the baseline levels of sIL-6ST and the response to IL-17/IL-17R inhibitors were found (Table 2). No differences between responders and non-responders were found in the baseline levels of TNF-α, IL-6, and sIL-6R in treatment series with IL-17/IL-17R inhibitors. In both the adalimumab and IL-17/IL-17R inhibitor treatment groups, the IL-17A, IL-1β and IFN-γ levels in the majority of patients were below the lower limit of detection and they did not allow for further analysis.

Sub-analyses using PASI ≤ 4 at three months after treatment initiation as the definition of a response showed the same results regarding IL-6 in the adalimumab group. However, there were no differences between responders and non-responders in the baseline levels of sIL-6R. The level of TNF-α was lower at baseline in responders than in non-responders. There were no differences between responders and non-responders in baseline levels of IL-6, sIL-6R, sIL-6ST, or TNF-α in the patients treated with IL-17/IL-17R inhibitors (Table S6, Figures S5 and S6).

## 3. Discussion

In this study of patients initiating biologics for treatment of psoriasis, the blood cell biomarker NLR was lower at baseline in responders than in non-responders to treatment with TNF-α inhibitors. For patients treated with adalimumab, we found lower levels of IL-6 at baseline in responders than in non-responders. Similar results were observed in the subgroup of bio-naive patients. The NLR has been reported to be higher in patients with psoriasis than in healthy controls [11]. Moreover, NLR has been shown to independently predict cardiovascular risk and all-cause mortality [16], and it may be a potential biomarker of cardiovascular disease in patients with psoriasis [13,14,15]. Additionally, treatment with biologics decreases the NLR levels in patients with psoriasis, and NLR has been proposed as a useful biomarker to monitor the disease course during treatment with biologics [17,18,19,20]. In this study, we found that NLR was lower at baseline in responders than in non-responders to treatment with TNF-α inhibitors, suggesting that this biomarker might also be predictive of the treatment response. Indeed, similar findings have been reported for NLR and PLR in patients with rheumatoid arthritis [21]. In the sub-analyses where the definition of a response was the achievement of PASI ≤ 4, we did not observe a difference in NLR at baseline between responders and non-responders in any of the treatment groups. However, with this less restrictive definition of treatment response, the number of non-responders was low (Tables S3 and S4) and a PASI ≤ 2 response is clearly more relevant in the clinical setting with the treatment options available today [22]. Aiming for a treatment response of a PASI ≤ 2 is also supported by results showing that a good treatment response with a low PASI the first six months after treatment initiation leads to fewer flare ups and improved drug survival over time [23].

We have previously found an association between low levels of IL-6 and TNF-α at baseline and a response to brodalumab in patients with psoriasis [24]. In the current study, we found similar results for IL-6 in patients treated with adalimumab, indicating that a lower level of IL-6 at baseline may favor a response to TNF-α inhibitors. IL-6 plays a crucial role in T_h_17 cell differentiation [25], suggesting that a low concentration of IL-6 corresponds to a relatively low number of T_h_17 cells. In rheumatoid arthritis, a high baseline frequency of circulating T_h_17 cells has been associated with a lack of response to TNF-α inhibitors [26]. In the current study, however, patients treated with brodalumab, an IL-17R inhibitor, were few (*n* = 12), and they grouped with patients treated with the IL-17 inhibitors, secukinumab and ixekizumab, which may contribute to the discrepancy with our previous results. Moreover, the current patients did not undergo a wash-out period between treatments (as is the case in prospective clinical trials), which could have impacted the baseline levels of blood cytokines.

Interestingly, we found that baseline sIL-6ST levels were higher in responders to IL-17/IL-17R inhibitors than in non-responders in the initial analysis; however, this was not significant in the logistic regression analysis. The connection between the IL-6 signaling pathway and IL-17 production is complex [27], but it is possible that the ratio of soluble to cell-bound IL-6R plays a role. Our results regarding the blood cytokines in the IL-17/IL-17R inhibitor group should be interpreted with caution due to the small sample size and low proportion of non-responders.

In the sub-analysis using a PASI ≤ 4 as the response definition, we found that levels of TNF-α were also lower at baseline in responders than in non-responders to adalimumab therapy. We have previously found similar results for patients treated with the IL-17R inhibitor brodalumb [24]. However, in this sub-analysis using PASI ≤ 4 as the treatment response, there were few non-responders, and the results should be interpreted with caution.

In general, across the different treatment groups, we observed a tendency for a higher BMI in non-responders than responders, which is in line with the existing literature describing that a high BMI is associated with a decreased chance of a favorable treatment response [6,7,8]. However, our results did not seem to be affected by BMI, as adjustments for this factor in the logistic regression analysis did not change our results.

Today, several effective biologics targeting different critical steps in the inflammatory process are available. Clinically, there is a large individual variation in response to biologics among patients with psoriasis, and the relative role of different cytokines in the disease pathogenesis is likely to differ between patients. In the future, personalized medicine based on predictive biomarkers may decrease the time to reach skin clearance and thereby modify the disease course and burden in line with the likely importance of early aggressive intervention in psoriasis [28].

Few studies have investigated biomarkers of systemic inflammation as predictive biomarkers of treatment response to biologics in patients with psoriasis. The focus on biomarkers that can be obtained from standard blood tests and the stratification of patients according to their bio-naivety are among the strengths of our study. Moreover, only patients with psoriasis vulgaris were included, and PASI was objectively determined by the treating physicians. Limitations include the pooling of patients treated with different biological drugs into classes according to which pathway the drugs target, as intra-class differences are possible. Moreover, the study was based on a limited sample size (especially for the IL-17/IL-17R inhibitor group), and the levels of many of the investigated cytokines were below the limits of quantification of the employed assays.

The findings of this study suggest that some biomarkers of systemic inflammation can be used as predictors of the response to treatment with biologics. Notably, NLR and IL-6 were found to be lower at baseline in bio-naive responders than bio-naive non-responders to TNF-α inhibitors. Larger studies focusing on the intra-class as well as inter-class differences of responses to biologics in psoriasis are warranted.

## 4. Materials and Methods

### 4.1. Patients and Data Sources

The study was conducted at the Department of Dermatology and Allergy, Herlev, and Gentofte Hospital, Denmark, and was approved by the Ethics Committee of the Capital Region of Denmark (H-19036920), and the Danish Data Protection Agency (P-2019-285). All patients gave informed consent. The study included patients with plaque psoriasis initiating treatment with biologics at our department from February 2020 to July 2022. Standard blood tests were performed for all patients before initiation of treatment. An additional blood sample was collected and kept at −80 °C until future analysis. Demographic data regarding sex, age, BMI, and smoking, and clinical data including psoriasis area and severity index (PASI), concomitant psoriatic arthritis (PsA), bio-naivety, and treatment start and stop dates were collected in the clinic. Results of standard blood tests were obtained from the Department of Clinical Biochemistry, Herlev, and Gentofte Hospital, Denmark.

Patients receiving any type of biologics were included in the analyses if a PASI was registered three months (±45 days) after treatment initiation and if the treatment start date could be matched with blood sample data obtained up to 45 days before treatment initiation. If a patient had two separate treatment series, both were included. Patients did not undergo a treatment washout before inclusion in the study. To exclude the impact of previous treatment with biologics, sub-analyses including only patients who were bio-naive were performed. Baseline cytokines and their association with response were analyzed for a subgroup of patients initiating treatment with adalimumab (TNF-α inhibitor), brodalumab (IL-17R inhibitor), ixekizumab (IL-17 inhibitor), or secukinumab (IL-17 inhibitor) with available baseline blood samples.

### 4.2. Analyses of Blood Cell Biomarkers and Cytokines at Baseline

Blood cell biomarkers of inflammation included NLR, PLR, and SII, and these were calculated using the following equations, as performed previously [11,29]:

NLR = neutrophils/lymphocytes, PLR = platelets/lymphocytes, and SII = (platelets × neutrophils)/lymphocytes.

For cytokine analyses, we used serum samples, which were stored at −80 °C until analysis. The samples were analyzed at Institute for Inflammation Research, Rigshospitalet, Denmark. The concentrations of IL-1β, IL-17A, TNF-α, IFN-γ, and IL-6 were measured using Luminex Performance assays (Bio-Techne Ltd., Abingdon, UK). Concentrations of the soluble forms of the IL-6 receptor (sIL-6R) and soluble IL-6 signal transducer (sIL-6ST) were measured with in-house developed Luminex assays. To ensure analytical quality, measured values were validated with control serum samples and partial duplicate analyses. The lower limits of detection were 1.0 pg/mL for IL-1β, 2.5 pg/mL for IL-17A, 1.1 pg/mL for TNF-α, 1.9 pg/mL for IFN-γ, 0.7 pg/mL for IL-6, 0.2 ng/mL for sIL-6R, and 1.0 ng/mL for sIL-6ST.

### 4.3. Definition of Treatment Response

Based on the registered PASI three months after treatment initiation, all included patients were divided into responders and non-responders. The predefined criterion for clinical response was the achievement of PASI ≤ 2 three months after initiation of treatment. Sub-analyses where the response definition was changed to PASI ≤ 4 after three months of treatment were also performed. An absolute PASI ≤ 2 and PASI ≤ 4 have been shown to correspond to a PASI90 and PASI75, respectively [23,30].

### 4.4. Statistical Analysis

Data distributions were assessed by quantile–quantile plots. Non-normally distributed data are reported as medians and interquartile ranges (IQRs). Wilcoxon–Mann–Whitney tests were used for statistical inference between responders vs. non-responders in analyses of blood cell biomarkers and cytokines. Significant findings from the Wilcoxon–Mann–Whitney tests were further tested by logistic regression analysis, where the dependent variable was response to treatment and the independent variable was the different inflammatory biomarkers. The logistic regression included adjustments for BMI, smoking status, and baseline PASI, which are factors previously shown to affect the response to biologics in patients with psoriasis [6,7,8]. All analyses were performed in RStudio with R version 4.1.3.

## Figures and Tables

**Table 1:** Baseline characteristics and levels of blood cell biomarkers of inflammation.

	Responders	Non-Responders	*p* Value	Bio-Naive, Responders	Bio-Naive, Non-Responders	*p* Value
TNF-α inhibitors *						
*n*	73	58	-	60	48	-
Age, years, median (IQR)	44 (32–52)	46 (32–60)	-	44 (33–52)	49 (32–60)	-
Sex, female, *n* (%)	21 (29)	18(31)	-	18 (30)	13 (27)	-
BMI, median (IQR)	26.6 (24.0–30.9) ^a^	29.4 (24.4–34.3)	-	26.8 (24.0–30.3)	28.9 (24.4–33.9)	-
Ever-smokers, *n* (%)	33 (45) ^a^	34 (39)	-	30 (50)	27 (56)	-
PsA, *n* (%)	13 (18) ^b^	18 (31) ^d^	-	11 (18) ^c^	14 (29)	-
Baseline PASI, median (IQR)	6.9 (4.6–12.0) ^e^	(9.8 (5.6–12.6) ^a^	-	7.9 (5.5–12.5) ^a^	10 (5.4–12.7) ^a^	-
Concomitant MTX treatment, *n* (%)	5 (7)	5 (9)	-	5 (8)	5 (10)	-
Lymphocytes, 10^9^/L, median (IQR)	1.7 (1.3–2.2) ^f^	1.7 (1.3–2.3) ^h^	-	1.7 (1.3–2.2) ^g^	1.7 (1.3–2.1) ^e^	-
Neutrophils, 10^9^/L, median (IQR)	3.7 (3.2–4.6) ^f^	4.4 (3.5–5.6) ^h^	-	3.6 (3.1–4.7) ^g^	4.5 (3.6–5.6) ^e^	-
Platelets, 10^9^/L, median (IQR)	250.0 (209.5–295.8) ^a^	261.0 (217.0–313.0) ^a^	-	253.0 (210.0–297.5) ^a^	261.0 (215.5–321.0) ^a^	-
NLR, median (IQR)	2.1 (1.7–2.9) ^f^	2.5 (1.9–3.5) ^h^	0.04	2.2 (1.7–2.7) ^g^	2.5 (2.0–3.8) ^e^	0.04
PLR, median (IQR)	148.2 (124.7–174.5) ^f^	148.2 (113.9–197.7) ^g^	0.79	151.8 (126.7–178.5) ^g^	151.3 (121.7–201.4) ^h^	0.55
SII, 10^9^/L, median (IQR)	552.7 (400.7–726.4) ^f^	653.6 (418.4–967.5) ^g^	0.08	552.7 (414.8–775.6) ^g^	689.0 (502.6–1060.0) ^h^	0.05
IL-23/IL-12/23 inhibitors **						
n	29	21	-	25	10	-
Age, years, median (IQR)	38 (23–45)	50 (33–55)	-	38 (23–45)	42 (30–50)	-
Sex, female, n (%)	11 (38)	5 (24)	-	10 (40)	2 (20)	-
BMI, median (IQR)	25.1 (22.0–30.4)	28.6 (27.3–32.6)	-	25.3 (22.4–30.4)	28.4 (28.0–29.9)	-
Ever-smokers, n (%)	16 (55)	11 (25)	-	12 (48)	4 (40)	-
PsA, n (%)	3 (10) ^d^	5 (24)	-	2 (8) ^i^	1 (10)	-
Baseline PASI, median (IQR)	9.9 (7.3–11.0)	10.2 (6.6–13.6)	-	9.6 (7.3–10.5)	8.1 (6.5–11.8)	-
Concomitant MTX treatment, n (%)	4 (14)	2 (9)	-	4 (16)	1 (10)	-
Lymphocytes, 10^9^/L, median (IQR)	1.6 (1.3–2.0) ^a^	1.6 (1.3–2.0) ^e^	-	1.5 (1.3–2.0)	1.6 (1.5–1.7) ^a^	-
Neutrophils, 10^9^/L, median (IQR)	3.9 (3.1–5.6) ^a^	4.3 (3.2–5.7) ^e^	-	3.9 (3.0–5.3)	3.3 (2.5–4.6) ^a^	-
Platelets, 10^9^/L, median (IQR)	262.0 (215.0–341.0)	255.0 (220.0–285.0)	-	249.0 (212.0–278.0)	252.5 (227.0–262.5)	-
NLR, median (IQR)	2.4 (2.0–3.0) ^a^	2.5 (1.8–3.5) ^e^	0.96	2.4 (1.9–2.6)	1.9 (1.6–3.5) ^a^	0.54
PLR, median (IQR)	149.0 (134.3–204.3) ^a^	159.0 (116.0–197.3) ^e^	0.82	148.7 (121.4–179.2)	159.0 (141.9–179.5) ^a^	0.59
SII, 10^9^/L, median (IQR)	600.5 (517.8–730.3) ^a^	554.1 (403.0–1037.4) ^e^	0.76	584.3 (503.9–678.7)	492.3 (363.8–970.6) ^a^	0.47
IL-17/IL-17R inhibitors ***						
*n*	54	11	-	23	1	-
Age, years, median (IQR)	44 (31–57)	54 (47–62)	-	41 (31–49)	-	-
Sex, female, *n* (%)	21 (39)	4 (36)	-	8 (35)	-	-
BMI, median (IQR)	26.1 (22.8–29.5)	34.7 (26.9–40.7)	-	23.8 (21.3–28.5)	-	-
Ever-smokers, *n* (%)	22 (41)	7 (64)	-	9 (39)	-	-
PsA, *n* (%)	19 (35) ^d^	2 (18) ^e^	-	6 (26) ^i^	-	-
Baseline PASI, median (IQR)	9.2 (7.0–11.4)	8.4 (6.2–11.2)	-	10.5 (7.5–13.8)	-	-
Concomitant MTX treatment, *n* (%)	3 (5)	0 (0)	-	3 (12)	-	-
Lymphocytes, 10^9^/L, median (IQR)	1.8 (1.4–1.9)	1.8 (1.7–2.2) ^g^	-	1.8 (1.4–2.0)	-	-
Neutrophils, 10^9^/L, median (IQR)	4.0 (3.1–4.8)	3.4 (2.8–6.0) ^g^	-	4.4 (3.3–4.9)	-	-
Platelets, 10^9^/L, median (IQR)	246.8 (208.0–294.0)	271.5 (269.0–288.0) ^a^	-	247.0 (215.0–294.0)	-	-
NLR, median (IQR)	2.3 (1.9–2.8)	2.1 (1.7–2.4) ^g^	0.28	2.5 (2.0–3.2)	-	-
PLR, median (IQR)	141.3 (108.1–185.4)	152.0 (120.2–164.8) ^g^	0.87	143.3 (121.5–184.9)	-	-
SII, 10^9^/L, median (IQR)	569.4 (446.3–745.7)	625.6 (476.2–714.2) ^g^	0.85	701.6 (463.8–789.9)	-	-

*p* value indicates the result of Wilcoxon–Mann–Whitney test. * TNF-α inhibitors included adalimumab (*n* = 125), infliximab (*n*= 3), certolizumab pegol (*n* = 2), and etanercept (*n* = 1). ** IL-23/IL-12/23 inhibitors included guselkumab (*n* = 10), risankizumab (*n* = 3), and ustekinumab (*n* = 37). *** IL-17/IL-17R inhibitors included secukinumab (*n* = 31), ixekizumab (*n* = 21), and brodalumab (*n* = 13). ^a^ Missing data (*n* = 1), ^b^ missing data (*n* = 19), ^c^ missing data (*n* = 17), ^d^ missing data (*n* = 9), ^e^ missing data (*n* = 2), ^f^ missing data (*n* = 5), ^g^ missing data (*n* = 4), ^h^ missing data (*n* = 3), and ^i^ missing data (*n* = 8). TNF, tumor necrosis factor; BMI, body mass index; PsA, psoriatic arthritis; PASI, psoriasis area and severity index; MTX, methotrexate; IL, interleukin; NLR, neutrophil-to-lymphocyte ratio; PLR, platelet-to-lymphocyte ratio; and SII; systemic immune–inflammation index.

**Table 2:** Results of logistic regression analysis.

**TNF-α Inhibitor Treatment Group**				
	**Responder** **OR (95% CI)**	**Responder** **OR (95% CI)** Adjusted *	**Bio-Naive,** **Responder** **OR (95% CI)**	**Bio-Naive,** **Responder** **OR (95% CI)** Adjusted *
NLR.	0.690 (0.487–0.943)	0.687 (0.479–0.952)	0.663 (0.450–0.931)	0.650 (0.436–0.924)
IL-6 (pg/mL)	0.720 (0.473–0.961)	0.716 (0.469–0.964)	0.578 (0.303–0-904)	0.528 (0.267–0.857)
sIL-6R (ng/mL)	0.968 (0.928–1.01)	0.968 (0.928–1.01)	0.961 (0.917–1.00)	0.963 (0.919–1.00)
**IL-17/IL-17R Inhibitor Treatment Group**				
	**Responder** **OR (95% CI)**	**Responder** **OR (95% CI)** Adjusted *	**-**	**-**
sIL-6ST (ng/mL)	1.04 (1.00–1.10)	1.04 (1.00–1.12)	-	-

* Adjusted for baseline PASI, BMI, and smoking status. TNF, tumor necrosis factor; NLR, neutrophil-to-lymphocyte ratio BMI, body mass index; PASI, psoriasis area and severity index; IL, interleukin; sIL-R, soluble interleukin 6 receptor; and sIL-6ST, soluble interleukin 6 signal transducer.

**Table 3:** Baseline characteristics and cytokine levels.

	Responders	Non-Responders	*p* Value	Bio-Naive, Responders	Bio-Naive, Non-Responders	*p* Value
Adalimumab						
*n*	35	27	-	31	24	-
Age, years, median (IQR)	43 (34–54)	54 (33–60)	-	42 (34–53)	51 (33–60)	-
Sex, female, *n* (%)	11 (31)	7 (26)	-	10 (32)	6 (25)	-
BMI, median (IQR)	28.2 (24.6–31.3)	27.7 (24.4–34)	-	28.3 (25.2–32.0)	28.5 (25.3–34.7)	-
Ever-smokers, *n* (%)	16 (46)	14 (52) ^a^	-	14 (45)	12 (50)	-
PsA, *n* (%)	5 (14)	7 (26)	-	4 (13)	6 (25)	-
Baseline PASI, median (IQR)	8.4 (4.2–13.8)	9.6 (6.0–12.0)	-	10.0 (5.3–14.0)	9.7 (6.2–11.9)	-
Concomitant MTX treatment, *n* (%)	1 (3)	1 (4)	-	1 (3)	1 (4)	-
TNF-α, pg/mL, median (IQR)	3.94 (2.90–5.15) ^b^	5.04 (1.83–7.42) ^d^	0.32	3.89 (2.83–5.09) ^c^	4.87 (1.85–7-69) ^d^	0.28
IL-6, pg/mL, median (IQR)	0.99 (0.42–1.48) ^c^	1.62 (0.96–2.41) ^e^	0.02	0.99 (0.42–1.42) ^c^	1.63 (1.10–2.36) ^e^	0.01
sIL-6ST, ng/mL, median (IQR)	123.34 (97.67–143.30)	123.36 (103.72–159.04)	0.37	123.34 (95.74–139.44)	130.44 (105.73–157.99)	0.18
sIL-6R, ng/mL, median (IQR)	30.11 (26.31–35.84)	39.63 (30.71–47.17)	0.06	30.11 (25.64–34.15)	39.83 (31.67–49.30)	0.03
IL-17/IL-17R inhibitors *						
*n*	17	7	-	0	0	-
Age, years, median (IQR)	52 (41–59)	61 (52–69)	-	-	-	-
Sex, female, *n* (%)	6 (35)	3 (43)	-	-	-	-
BMI, median (IQR)	28.6 (26.6–33.7)	31.6 (24.1–39.8)	-	-	-	-
Ever-smokers, *n* (%)	7 (41)	1 (14)	-	-	-	-
PsA, *n* (%)	5 (29)	1 (14)	-	-	-	-
Baseline PASI, median (IQR)	7.2 (5.3–8.9)	8.4 (5.0–10.3)	-	-	-	-
Concomitant MTX treatment, *n* (%)	0 (0)	0 (0)	-	-	-	-
TNF-α, pg/mL, median (IQR)	4.57 (3.07–9.24) ^e^	2.24 (1.33–3.83)	0.11	-	-	-
IL-6, pg/mL, median (IQR)	2.16 (0.64–6.02) ^e^	1.04 (0.84–1.24)	0.42	-	-	-
sIL-6ST, ng/mL, median (IQR)	128.12 (110.85–159.21)	104.60 (101.64–106.10)	0.02	-	-	-
sIL-6R, ng/mL, median (IQR)	32.62 (27.29–37.61)	37.22 (32.55–39.77)	0.29	-	-	-

*p* value indicates the result of Wilcoxon–Mann–Whitney test. * IL-17/IL-17R inhibitors included secukinumab (*n* = 1), ixekizumab (*n* = 11), and brodalumab (*n* = 12). ^a^ Missing data (*n* = 1), ^b^ samples below detection limit (*n* = 4), ^c^ samples below detection limit (*n* = 2), ^d^ samples below detection limit (*n* = 3), and ^e^ samples below detection limit (*n* = 1). TNF, tumor-necrosis factor; BMI, body mass index; PsA, psoriatic arthritis; PASI, psoriasis area and severity index; MTX, methotrexate; IL, interleukin; sIL-6ST, soluble interleukin-6 signal transducer; and sIL-6R, soluble interleukin-6 receptor.

## Data Availability

The data presented in this manuscript are available on request from the corresponding author.

## Supplementary Materials

The following supporting information can be downloaded at: https://www.mdpi.com/article/10.3390/ijms24076111/s1.
