# Supplementary material for: Blood Cell Biomarkers of Inflammation and Cytokine Levels as Predictors of Response to Biologics in Patients with Psoriasis
Source: Int J Mol Sci. 2023 Mar 24;24(7):6111. doi: 10.3390/ijms24076111 (PMC10094459; doi:10.3390/ijms24076111)
Supplement: Supplementary file 1 [file ijms-24-06111-s001.zip › ijms-2264917-supplementary.pdf]

Figure S1: Overview of included patients and treatment series in the analysis of blood cell biomarkers

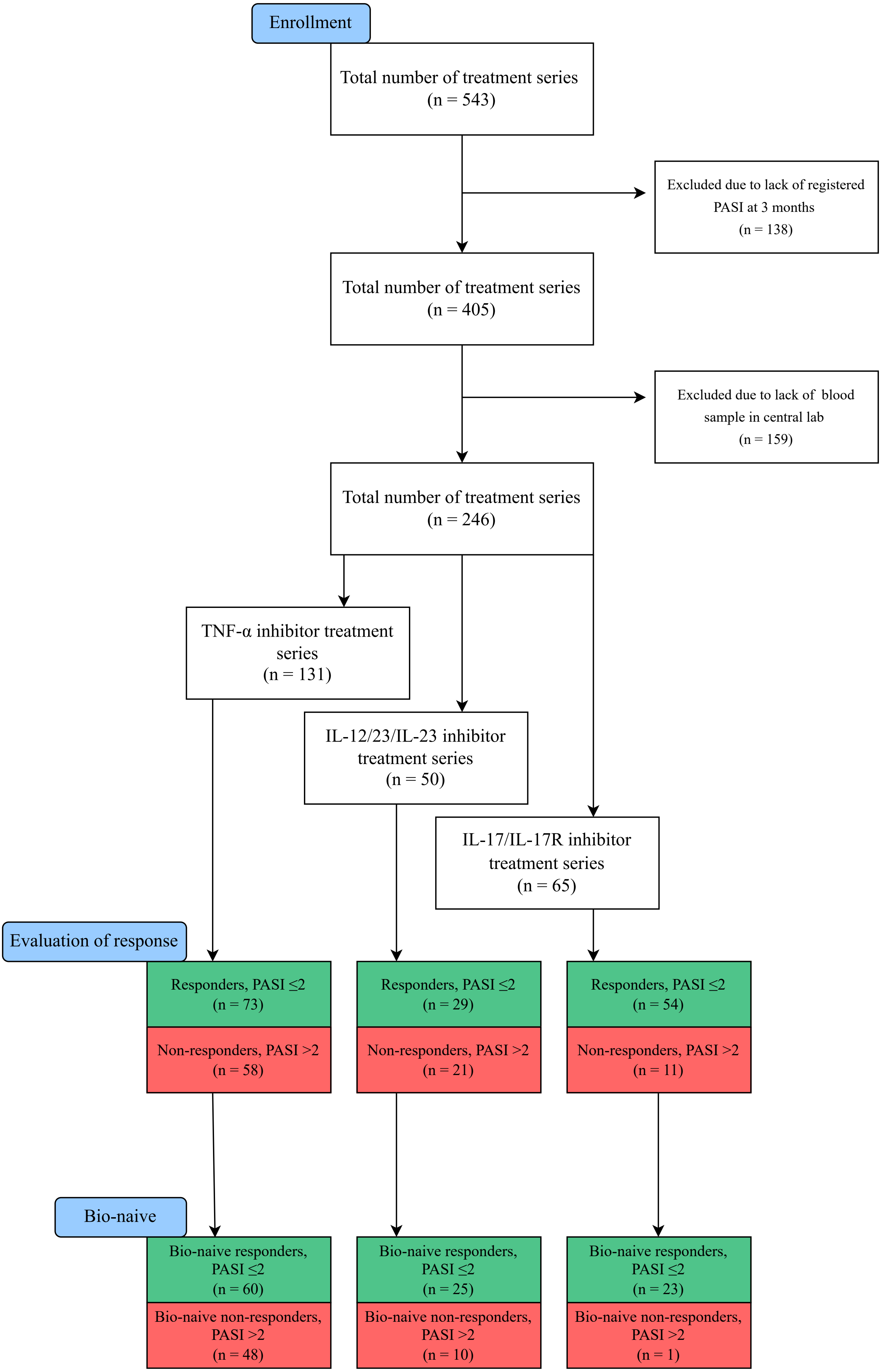

Figure S2: Overview of included patients and treatment series in the analysis of blood cytokines

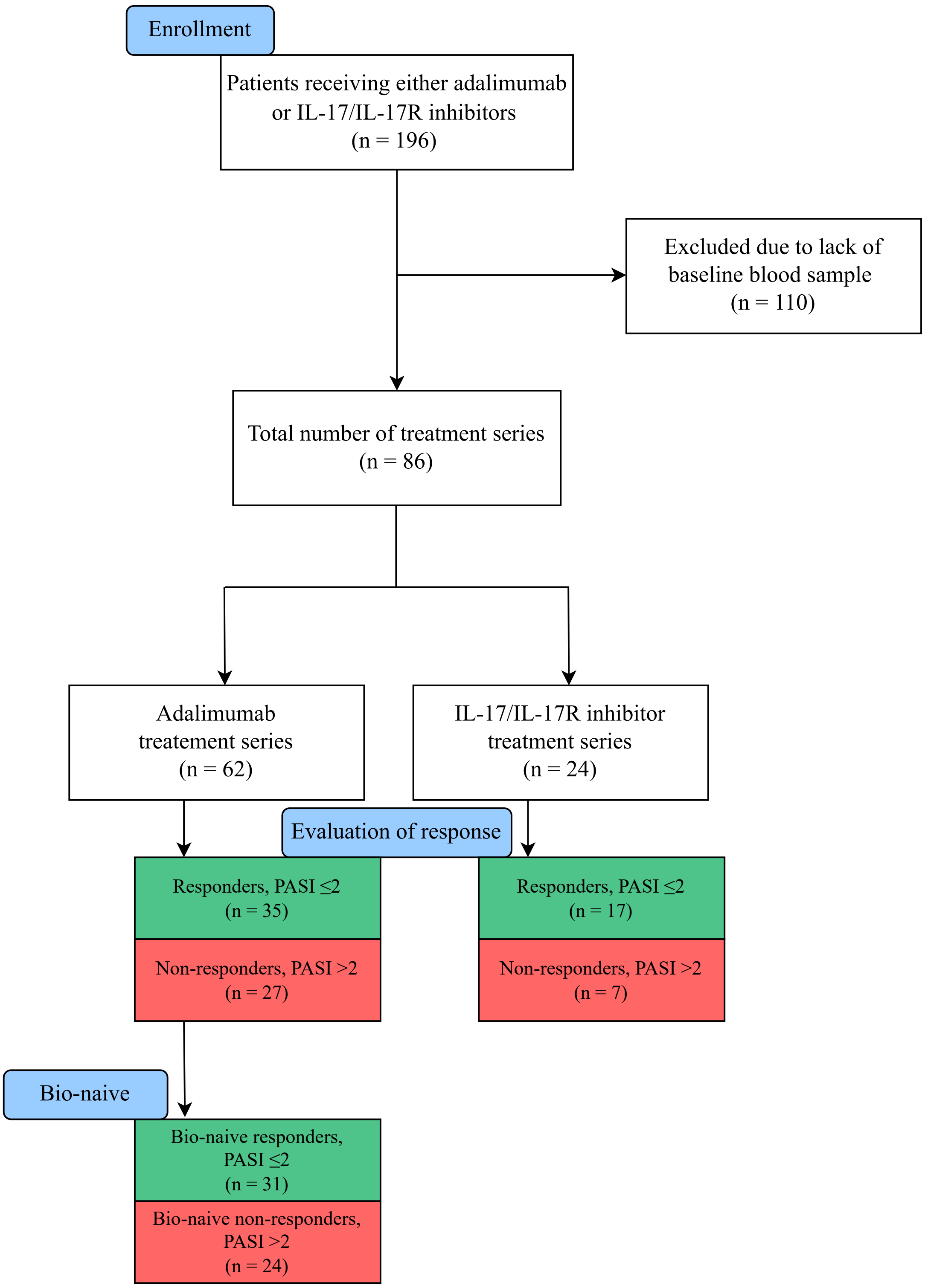

Table S1: Treatments included in the analysis of blood cell biomarkers of inflammation.

	**TNF-α inhibitors**	**IL-17/IL-17R inhibitors**	**IL-23 inhibitors**	**IL-12/23 inhibitors**
Adalimumab	125			
Certolizumab pegol	2			
Etanercept	1			
Infliximab	3			
Secukinumab		31		
Ixekizumab		21		
Brodalumab		13		
Ustekinumab				37
Guselkumab			10	
Risankizumab			3	
**Total**	131	65	13	37

Table S2: Treatments included in the analysis of blood cytokines

	**TNF-α inhibitors**	**IL-17/IL-17R inhibitors**
Adalimumab	62	
Ixekizumab		11
Brodalumab		12
Secukinumab		1
**Total**	62	24

Table S3: Blood cell biomarkers of inflammation, numbers of responders and non-responders for different PASI definitions of response to each type of biologics

Response definition	**PASI≤2**		**PASI≤4**	
	Responders	Non-responders	Responders	Non-responders
**TNF-α inhibitors**				
Adalimumab	71	54	103	22
Infliximab	0	3	2	1
Certolizumab pegol	1	1	1	1
Etanercept	1	0	1	0
Total	*73*	*58*	*107*	*24*
**IL-12/23 inhibitors**				
Ustekinumab	25	12	31	6
Total	*25*	*12*	*31*	*6*
**IL-23 inhibitors**				
Guselkumab	4	6	5	5
Risankizumab	0	3	1	2
Total	*4*	*9*	*6*	*7*
**IL-17 inhibitors**				
Secukinumab	29	2	31	0
Ixekizumab	14	7	19	2
Total	*33*	*9*	*50*	*2*
**IL-17R inhibitors**				
Brodalumab	11	2	12	1
Total	*11*	*2*	*12*	*1*

Table S4: Blood cytokines, numbers of responders and non-responders for different PASI definitions of response to each type of biologics

Response definition	**PASI≤2**		**PASI≤4**	
	Responders	Non-responders	Responders	Non-responders
**TNF-α inhibitors**				
Adalimumab	35	27	49	13
**IL-17 inhibitors**				
Secukinumab	1	0	1	0
Ixekizumab	6	5	10	1
**IL-17R inhibitors**				
Brodalumab	10	2	11	1

Table S5: Baseline characteristics and levels of blood cell biomarkers of inflammation. Response defined as a PASI ≤4.

	Responders	Bio-naive, responders	Non-responders	Bio-naive, non-responders
**TNF-α inhibitors***				
n	107	90	24	18
Age, years, median (IQR)	44 (32-54)	44 (32-56)	45 (33-61)	47 (37-64)
Sex, female, n (%)	31 (29)	26 (29)	8 (33)	5 (28)
BMI, median (IQR)	26.9 (24.3-31.3)[a]	26.9 (34.1-31.1)	31.0 (24.6-34.5)	30.0 (25.1-34.3)
Ever-smokers, n (%)	50 (47)[a]	46 (51)	17 (71)	11 (61)
PsA, n (%)	22 (21)[b]	18 (20)[c]	9 (38)[d]	7 (39)[e]
Baseline PASI, median (IQR)	7.9 (4.8-11.8)[e]	8.1 (5.1-12.3)	10.8 (8.5-12.9)	11.0 (8.8.-12.8)
Lymphocytes, 10^9/L, median (IQR)	1.7 (1.3-2.1)[f]	1.7 (1.3-2.1)[g]	1.7 (1.4-2.5)[a]	1.6 (1.3-2.3)
Neutrophils, 10^9/L, median (IQR)	3.9 (3.2-5.2)[f]	4.0 (3.2-5.2)[g]	4.2 (3.2-5.9)[a]	4.7 (3.0-5.9)
Platelets, 10^9/L, median (IQR)	252.0 (210.8-300.8)[a]	253.0 (210.0-301.0)[a]	261.0 (216.0-316.5)[a]	261.0 (218.0-387.0)[a]
NLR, median (IQR)	2.2 (1.8-3.1)[f]	2.2 (1.8-3.1)[g]	2.2 (1.6-3.49)[a]	2.5 (1.7-3.6)
PLR, median (IQR)	149.6 (123.5-176.5)[f]	150.3 (123.5-178.5)[g]	144.8 (95.1-201.3)[h]	185.0 (121.7-221.0)[a]
SII, 10^9/L, median (IQR)	580.7 (425.1-775.6)[f]	585.2 (436.6-802.6)[g]	524.7 (351.4-1161.5)[h]	742.8 (369.2-1374.7)[a]
**IL-23/IL-12/23 inhibitors****	37	30	13	5
n				
Age, years, median (IQR)	38 (25-50)	38 (24-46)	50 (35-56)	50 (36-50)
Sex, female, n (%)	12 (32)	11 (37)	4 (31)	1 (20)
BMI, median (IQR)	25.4 (22.5-30.4)	27.6 (23.4-30.2)	30.0 (27.3-34.9)	28.1 (25.1-30.0)
Ever-smokers, n (%)	18 (49)	14 (47)	9 (69)	3 (60)
PsA, n (%)	3 (8)[i]	2 (7)[j]	5 (38)	1 (20)
Baseline PASI, median (IQR)	9.3 (6.8-11.0)	9.0 (7.1-10.5)	12.6 (6.5-13.9)	10.2 (6.5-12.3)
Lymphocytes, 10^9/L, median (IQR)	1.7 (1.4-2.0)[h]	1.7 (1.4-2.0)[a]	1.5 (1.2-1.9)[a]	1.5 (1.4-1.5)
Neutrophils, 10^9/L, median (IQR)	4.1 (3.2-5.6)[h]	3.9 (3.0-5.6)[a]	4.3 (3.0-5.6)[a]	3.1 (2.5-3.3)
Platelets, 10^9/L, median (IQR)	262.0 (232.0-341.0)	253.0 (219.2-278.0)	251.0 (220.0-263.0)	220.0 (220.0-255.0)
NLR, median (IQR)	2.5 (1.9-3.3)[h]	2.4 (1.9-2.9)[a]	2.4 (1.8-3.4)[a]	1.9 (1.6-2.1)
PLR, median (IQR)	159.0 (139.2-206.0)[h]	148.8 (121.4-179.5)[a]	142.8 (98.8-184.1)[a]	143.8 (141.9-175.9)
SII, 10^9/L, median (IQR)	640.2 (513.1-797.2)[h]	584.3 (492.3-690.0)[a]	514.6 (356.2-1025.9)[a]	363.8 (333.5-545.2)
**IL-17/IL-17R inhibitors*****	62	24	3	0

n				
Age, years, median (IQR)	44 (32-58)	42 (31-51)	58 (55-67)	-
Sex, female, n (%)	24 (39)	8 (33)	1 (33)	-
BMI, median (IQR)	26.5 (23.1-31.5)	24.0 (21.3-29.1)	40.3 (37.8-45.0)	-
Ever-smokers, n (%)	26 (43)	9 (37)	3 (100)	-
PsA, n (%)	20 (32)[k]	6 (25)[j]	1 (33)	-
Baseline PASI, median (IQR)	9.0 (7.0-11.4)[a]	10.6 (7.5-13.6)	8.4 (6.8-11.7)	-
Lymphocytes, 10^9/L, median (IQR)	1.8 (1.4-1.9)[e]	1.7 (1.4-1.9)	2.5 (2.4-2.7)[a]	-
Neutrophils, 10^9/L, median (IQR)	4.0 (3.1-4.8)[e]	4.2 (3.2-4.8)	6.6 (6.4-6.9)[a]	-
Platelets, 10^9/L, median (IQR)	261.0 (214.0-294.0)[e]	252.5 (215.5-302.2)	259.0 (242.0-275.5)	-
NLR, median (IQR)	2.3 (1.9-2.8)[e]	2.4 (2.0-3.1)	2.7 (2.4-2.9)[a]	-
PLR, median (IQR)	143.3 (114.1-184.9)[e]	147.0 (122.8-196.0)	109.6 (105.5-113.6)[a]	-
SII, 10^9/L, median (IQR)	562.1 (443.2-721.9)[e]	702.7 (469.7-779.9)	730.7 (678.1-783.3)[a]	-

[*]TNF-α inhibitors included adalimumab (n=125), infliximab (n= 3), certolizumab pegol (n=2) and etanercept (n=1). [**]IL-23/IL-12/23 inhibitors included guselkumab (n=10), risankizumab (n=3) and ustekinumab (n=37). [***]IL-17/IL-17R inhibitors included secukinumab (n=31), ixekizumab (n=21) and brodalumab (n=13). [a] missing data (n = 1), [b] missing data (n = 23), [c] missing data (n = 20), [d] missing data (n = 5), [e] missing data (n = 3), [f] missing data (n = 7), [g] missing data (n = 6), [h] missing data (n = 2), [i] missing data (n = 9), [j] missing data (n=8), [k] missing data (n=11). TNF, tumor necrosis factor; BMI, body mass index; PsA, psoriatic arthritis; PASI, psoriasis area and severity index; IL, interleukin; NLR, neutrophil-to-lymphocyte ratio; PLR, platelet-to-lymphocyte ratio; SII; systemic immune-inflammation index.

Figure S3: Blood cell biomarkers at baseline in responders (PASI ≤4) and non-responders (PASI >4) treated with either tumor necrosis factor (TNF)-α inhibitors, interleukin (IL)-23/IL-12/23 inhibitors, or IL-17/IL-17R inhibitors A) Neutrophil-to-lymphocyte ratio (NLR). B) Platelet-to-lymphocyte ratio (PLR). C) Systemic immune-inflammation index (SII). Responders = psoriasis area and severity index (PASI) ≤4 three months after treatment start. Non-responders = PASI >4 three months after treatment start. Ns = non-significant. * $p < 0.05$.

Figure S4: Blood cell biomarkers at baseline in bio-naive responders (PASI ≤4) and non-responders (PASI >4) treated with either tumor necrosis factor (TNF)-α inhibitors or interleukin (IL)-23/IL-12/23 inhibitors. A) Neutrophil-to-lymphocyte ratio (NLR). B) Platelet-to-lymphocyte ratio (PLR). C) Systemic immune-inflammation index (SII). Responders = psoriasis area and severity index (PASI) ≤4 three months after treatment start. Non-responders = PASI >4 three months after treatment start. Ns = non-significant. * $p < 0.05$.

Figure S3

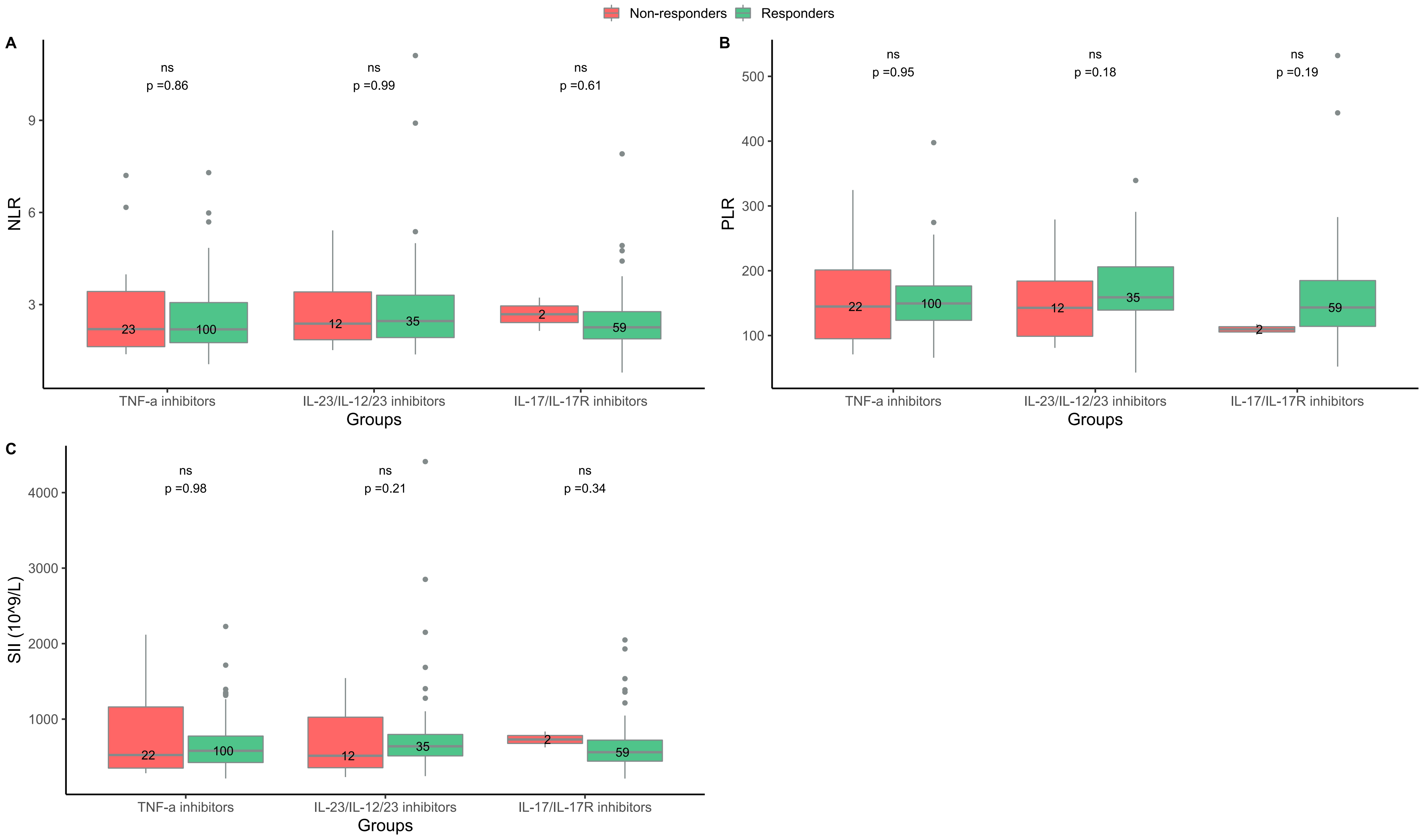

Non-responders
Responders
A
NLR
ns
p =0.86
ns
p =0.99
ns
p =0.61
9
6
3
23
100
12
35
2
59
TNF-a inhibitors
IL-23/IL-12/23 inhibitors
IL-17/IL-17R inhibitors
Groups
B
PLR
ns
p =0.95
ns
p =0.18
ns
p =0.19
500
400
300
200
100
22
100
12
35
2
59
TNF-a inhibitors
IL-23/IL-12/23 inhibitors
IL-17/IL-17R inhibitors
Groups
C
SII (10^9/L)
ns
p =0.98
ns
p =0.21
ns
p =0.34
4000
3000
2000
1000
22
100
12
35
2
59
TNF-a inhibitors
IL-23/IL-12/23 inhibitors
IL-17/IL-17R inhibitors
Groups

Figure S4

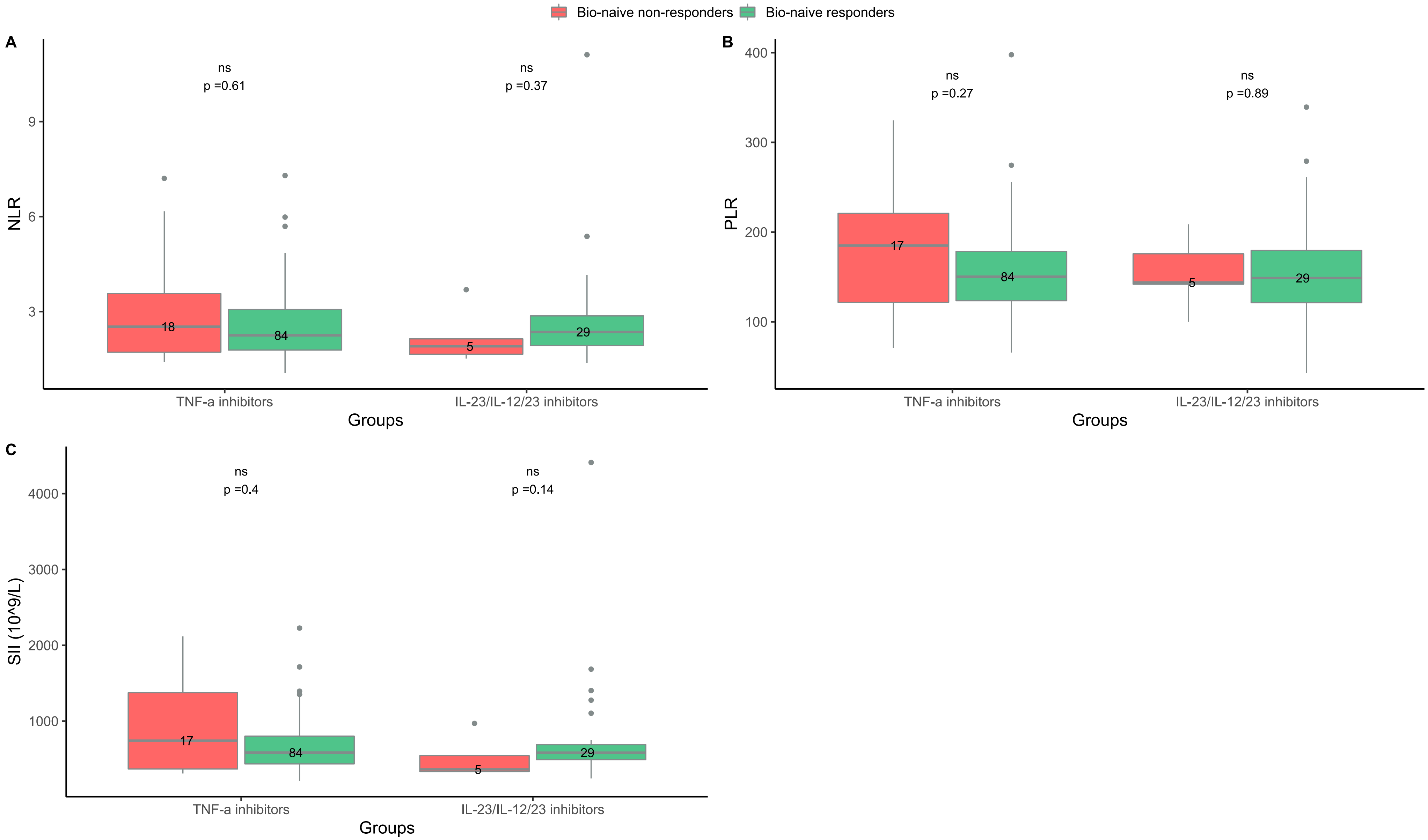
Bio-naive non-responders
Bio-naive responders
A
NLR
ns
p =0.61
ns
p =0.37
18
84
5
29
3
6
9
TNF-a inhibitors
IL-23/IL-12/23 inhibitors
Groups
B
PLR
ns
p =0.27
ns
p =0.89
17
84
5
29
100
200
300
400
TNF-a inhibitors
IL-23/IL-12/23 inhibitors
Groups
C
SII (10^9/L)
ns
p =0.4
ns
p =0.14
17
84
5
29
1000
2000
3000
4000
TNF-a inhibitors
IL-23/IL-12/23 inhibitors
Groups

Table S6: Baseline characteristics and cytokine levels. Response defined as an absolute PASI ≤4.

	Responders	Bio-naive, responders	Non-responders	Bio-naive, non-responders
**Adalimumab**				
n	49	43	13	12
Age, years, median (IQR)	43 (32-55)	42 (32-54)	56 (44-67)	55 (42-68)
Sex, female, n (%)	16 (33)	14 (33)	2 (15)	2 (17)
BMI, median (IQR)	27.7 (24.4-31.6)	28.2 (25.6-32.0)	30.3 (24.4-34.3)	30.0 (24.1-34.7)
Ever-smokers, n (%)	22 (46)[a]	19 (44)[a]	8 (62)	7 (58)
PsA, n (%)	7 (14)	6 (14)	5 (38)	4 (33)
Baseline PASI, median (IQR)	8.2 (4.5-11.9)	9.2 (4.9-13.8)	11.4 (9.4-12.6)	10.7 (9.1-12.4)
TNF-α, pg/mL, median (IQR)	3.89 (2.55-5.20)[b]	3.66 (2.55-5.20)[c]	6.62 (4.97-8.85)[d]	6.43 (4.87-9.35)[d]
IL-6, pg/mL, median (IQR)	1.01 (0.59-1.65)[d]	1.04 (0.63-1.64)[d]	1.86 (1.17-4.09)	1.78 (1.13-2.86)
sIL-6ST, ng/mL, median (IQR)	123.19 (96.36-147.56)	123.34 (96.91-146.86)	137.51 (116.79-152.51)	130.44 (114.78-149.03)
sIL-6R, ng/mL, median (IQR)	31.46 (26.92-43.06)	32.06 (26.31-43.99)	39.63 (32.03-45.64)	38.25 (31.96-42.94)
**IL-17/IL-17R inhibitors**[*]				
n	22	0	2	0
Age, years, median (IQR)	53 (42-60)	-	63 (57-69)	-
Sex, female, n (%)	9 (41)	-	0 (0)	-
BMI, median (IQR)	28.5 (24.6-33.3)	-	40.5 (38.5-42.4)	-
Ever-smokers, n (%)	14 (63)	-	2 (100)	-
PsA, n (%)	6 (27)	-	0 (0)	-
Baseline PASI, median (IQR)	7.1 (5.2-9.0)	-	11.8 (10.1-13.4)	-
TNF-α, pg/mL, median (IQR)	4.21 (2.74-9.06)[e]	-	1.88 (1.69-2.06)	-
IL-6, pg/mL, median (IQR)	1.04 (0.73-4.15)[e]	-	2.65 (2.00-3.20)	-
sIL-6ST, ng/mL, median (IQR)	115.50 (105.83-155.81)	-	126.80 (113.60-139.99)	-
sIL-6R, ng/mL, median (IQR)	33.34 (29.19-38.25)	-	27.25 (22.27-32.24)	-

[*]IL-17/IL-17R inhibitors included secukinumab (n=1), ixekizumab (n=11) and brodalumab (n=12). [a] Missing data (n=1), [b] samples below detection limit (n=4), [c] samples below detection limit (n=2), [d] samples below detection limit (n=3), [e] samples below detection limit (n=1). TNF, tumor-necrosis factor; BMI, body mass index; PsA, psoriatic arthritis; PASI, psoriasis area and severity index; IL, interleukin; sIL-6ST, interleukin-6 signal transducer; sIL-6R, interleukin-6 receptor.

Figure S5: Blood cytokine levels at baseline in responders (PASI ≤4) and non-responders (PASI >4) to treatment with adalimumab or interleukin (IL)-17/IL-17R inhibitors. A) Tumor necrosis factor-alpha (TNF)-α. B) Soluble interleukin-6 signal transducer (sIL-6ST). C) Interleukin-6 (IL-6). D) Soluble interleukin-6 receptor (sIL-6R). Responders = psoriasis area and severity index (PASI) ≤4 three months after treatment start. Non-responders = PASI >4 three months after treatment start. Ns = non-significant. * $p < 0.05$.

Figure S6: Blood cytokine levels at baseline in bio-naive responders (PASI ≤4) and non-responders (PASI >4) to treatment with adalimumab. A) Tumor necrosis factor-alpha (TNF)-α. B) Soluble interleukin-6 signal transducer (sIL-6ST). C) Interleukin-6 (IL-6). D) Soluble interleukin-6 receptor (sIL-6R). Responders = psoriasis area and severity index (PASI) ≤4 three months after treatment start. Non-responders = PASI >4 three months after treatment start. Ns = non-significant. * $p < 0.05$.

Figure S5

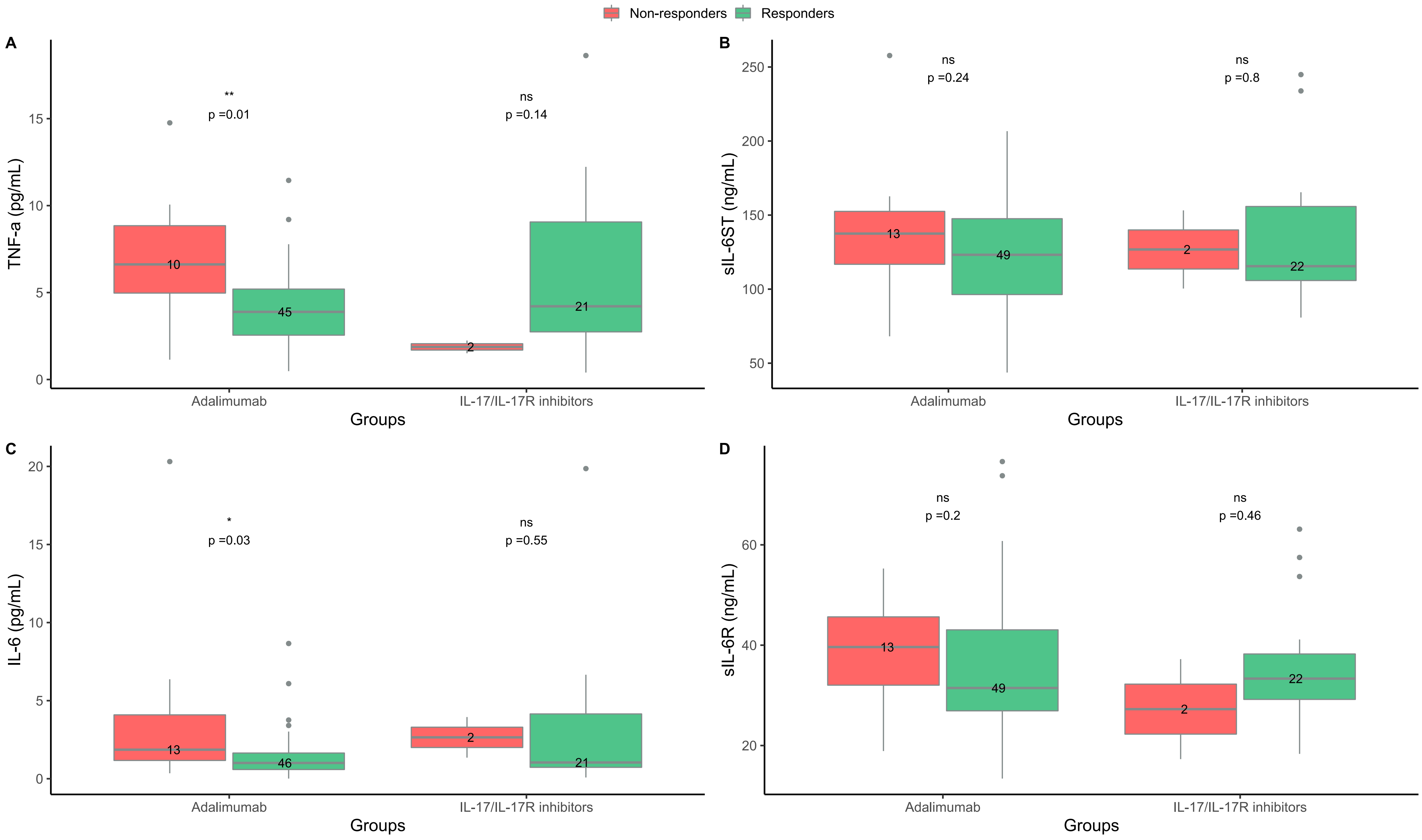

Non-responders
Responders
A
TNF-a (pg/mL)
**
p =0.01
ns
p =0.14
10
45
2
21
Adalimumab
IL-17/IL-17R inhibitors
Groups
B
sIL-6ST (ng/mL)
ns
p =0.24
ns
p =0.8
13
49
2
22
Adalimumab
IL-17/IL-17R inhibitors
Groups
C
IL-6 (pg/mL)
*
p =0.03
ns
p =0.55
13
46
2
21
Adalimumab
IL-17/IL-17R inhibitors
Groups
D
sIL-6R (ng/mL)
ns
p =0.2
ns
p =0.46
13
49
2
22
Adalimumab
IL-17/IL-17R inhibitors
Groups

Figure S6

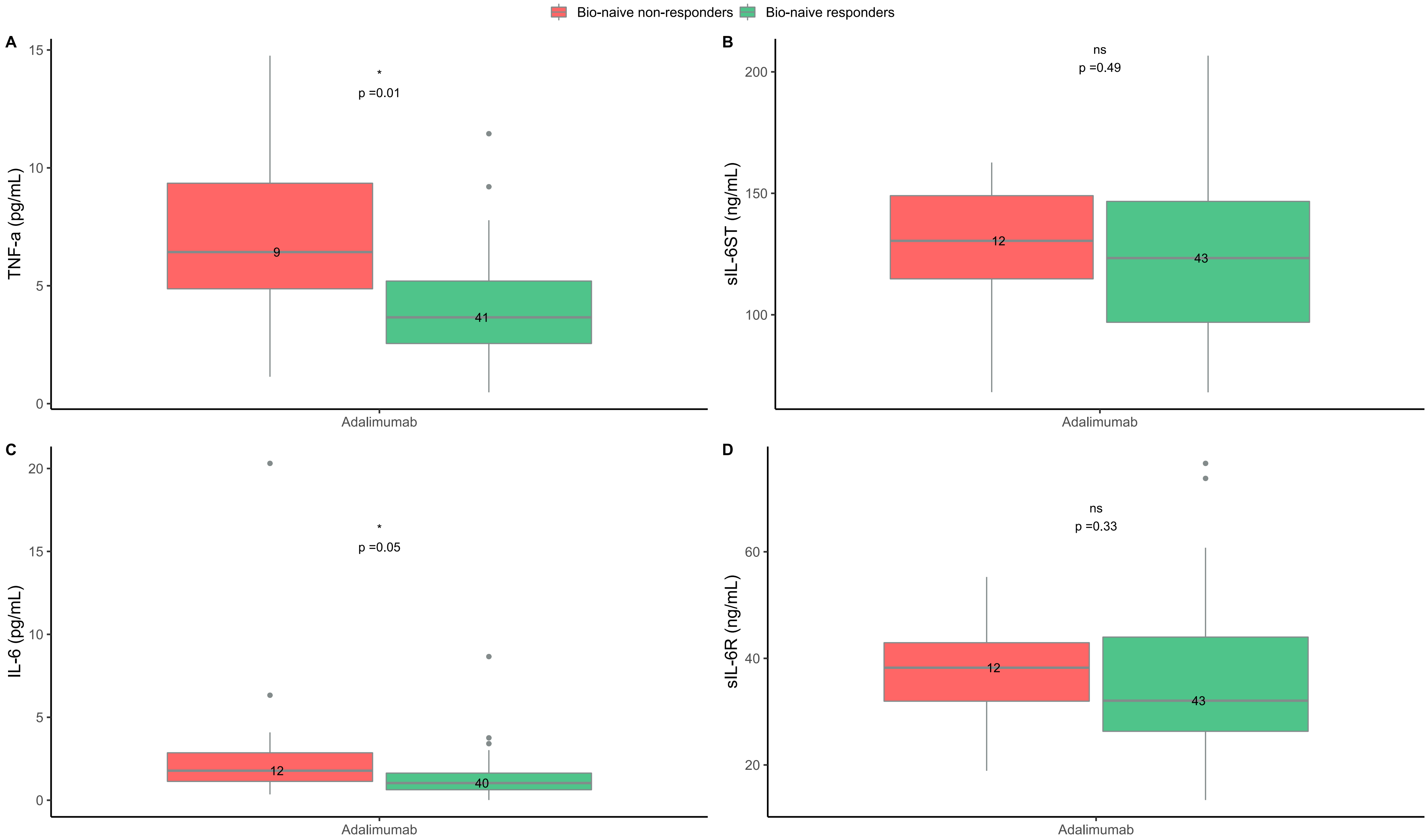

Bio-naive non-responders
Bio-naive responders
A
TNF-a (pg/mL)
15
10
5
0
*
p =0.01
9
41
Adalimumab
B
sIL-6ST (ng/mL)
200
150
100
ns
p =0.49
12
43
Adalimumab
C
IL-6 (pg/mL)
20
15
10
5
0
*
p =0.05
12
40
Adalimumab
D
sIL-6R (ng/mL)
60
40
20
ns
p =0.33
12
43
Adalimumab
